# Chip-scale optical airflow sensor

**DOI:** 10.1038/s41378-021-00335-1

**Published:** 2022-01-04

**Authors:** Yumeng Luo, Xiaoshuai An, Liang Chen, Kwai Hei Li

**Affiliations:** 1 School of Microelectronics, Southern University of Science and Technology, Shenzhen, 518055 China; 2 Engineering Research Center of Integrated Circuits for Next-Generation Communications, Ministry of Education, Southern University of Science and Technology, Shenzhen, 518055 China; 3 Engineering Research Center of Three Dimensional Integration in Guangdong Province, Southern University of Science and Technology, Shenzhen, 518055 China

**Keywords:** Electrical and electronic engineering, Optical sensors

## Abstract

Airflow sensors are an essential component in a wide range of industrial, biomedical, and environmental applications. The development of compact devices with a fast response and wide measurement range capable of in situ airflow monitoring is highly desirable. Herein, we report a miniaturized optical airflow sensor based on a GaN chip with a flexible PDMS membrane. The compact GaN chip is responsible for light emission and photodetection. The PDMS membrane fabricated using a droplet-based molding process can effectively transform the airflow stimuli into optical reflectance changes that can be monitored by an on-chip photodetector. Without the use of external components for light coupling, the proposed sensor adopting the novel integration scheme is capable of detecting airflow rates of up to 53.5 ms^−1^ and exhibits a fast response time of 12 ms, holding great promise for diverse practical applications. The potential use in monitoring human breathing is also demonstrated.

## Introduction

The velocity measurement and distribution analysis of airflow is of great significance in the fields of atmospheric environmental monitoring, aerodynamic studies, turbine inspection, navigation control, biomedical engineering, and so on^1–5^. Sensing devices employing the principles of thermoresistance^6–9^, piezoresistance^10–13^, electrical resistance^14–16^, capacitance^17^, magnetoelasticity^18^, and mechanoluminescence^19^ have been developed for airflow detection. However, the achievement of a sensor with both fast response and a wide measurement range remains an unsolved challenge. Recently, optical airflow sensors based on fiber optics have attracted substantial research interest due to their distinctive advantages, such as their lightweight, high sensitivity, and fast optical response^20,21^. To improve the sensitivity of sensors to optical changes induced by airflow, several advanced architectures, such as fiber Bragg gratings^22,23^, Fabry–Pérot interferometers^24,25^, microcantilevers^26^, and other nanostructures^27^, have been incorporated. Nevertheless, the reported fiber-optic sensing systems mostly involve the complex assembly of external components and require precise optical alignment to couple the light from a light source to a spectroscopic analyzer through optical fiber as the sensing medium. The bulky and complex configurations inevitably weaken the efficiency, robustness, and compactness of the system and limit its scope of applications. Furthermore, despite their intrinsic fast response based on optical means, the response time of the reported optical systems in response to airflow has not been investigated and presented.

Eliminating the external light-coupling components is a promising approach for realizing the miniaturization of optical airflow sensors. Considering the materials used to construct solid-state high-performance optical devices, GaN-based direct bandgap semiconductors and their alloys are excellent candidates because of their high efficiency, fast transient response, good physical and chemical stability, and long operational lifespan^28,29^. Recently, the monolithic integration of GaN-based optical and electronic components on a chip-scale platform has been demonstrated for on-chip visible light communication^30,31^, heart rate detection^32^, and illumination and imaging^33^. However, to date, reports on the use of integrated GaN-based devices for airflow detection remain extremely limited. The major limitation is that the existence of rigid and hard growth substrates, such as sapphire and silicon, limits the deformation of the device in response to airflow. The only report about a GaN-based airflow sensor employs a suspended GaN membrane that shows a detectable airflow rate of up to 2.779 ms^−1^ and a switch-on time of 1 s^34^. Although a bendable GaN film can be realized by the selective wet etching of silicon or laser lift-off of sapphire^35,36^, the use of a free-standing GaN film as a sensing medium responding to high airflow is less reliable due to its fragility. Moreover, structural deformation introduces unwanted strain in the GaN epitaxial layer, which degrades the internal quantum efficiency of the device^36^. Therefore, a highly flexible structure should be explored as an alternative sensing medium.

**Fig. 1:**
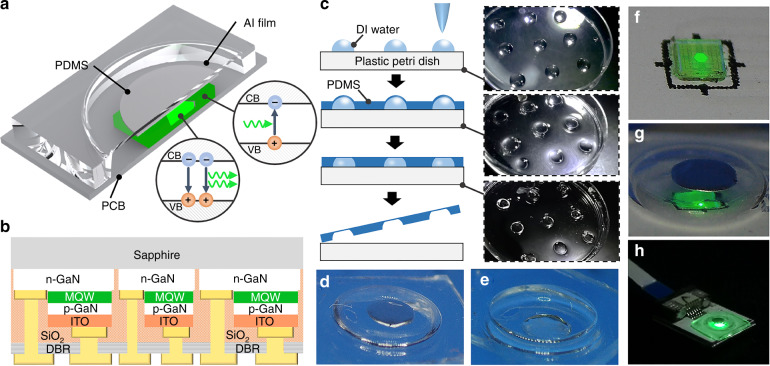
Structure of the optical airflow sensor. Schematic diagrams of the **a** proposed airflow sensor and **b** structural layout of the GaN chip. **c** Schematic illustrations of the fabrication process of the PDMS membrane and the corresponding optical images at different stages. Microphotographs of the **d** frontside and **e** backside of the PDMS membrane with Al film. Magnified image of the packaged chip **f** before and **g** after coverage of the PDMS membrane. **h** Optical image of the resultant airflow sensor

In this work, a novel compact integration of a GaN chip with a flexible PDMS membrane that enables the chip to sense airflow without the need for external light coupling components is proposed, as shown in Fig. 1a. The GaN chip with the dual functions of emission and detection is fabricated on a GaN/sapphire template through wafer-scale fabrication processes, while the PDMS membrane is formed by a low-cost droplet-based molding process. The electrical and optical properties of the on-chip device, as well as its ability to detect a wide range of airflow rates, are studied to confirm the effectiveness of the proposed integration scheme.

## Results and discussion

GaN chips are fabricated through wafer-scale microfabrication processes, including photolithography, etching, and deposition of metal and oxide layers. The structural layout of the GaN chip is schematically shown in Fig. 1b. The PDMS membrane structure is fabricated by a molding process using water droplets as a template, as depicted in Fig. 1c. With the highly sticky nature of the PDMS surface, the Al film can be adhered firmly to the PDMS membrane, as shown in Fig. 1d, e. The PDMS block is then fixed on the GaN chip by gluing the film edges with the PDMS gel, followed by a curing process. The packaged chip integrated with the PDMS membrane is shown in Fig. 1f**–**h. The electrical connection to the on-chip devices can be established through the Al printed circuit board (PCB) package; that is, the LED can be biased by a current source, and the photocurrent current can be read out by an ammeter.

The working principle of the proposed airflow sensor is shown in Fig. 1a. Under current injection, the LED emits light as the carriers confined in the InGaN active region recombine radiatively. The bottom distributed Bragg reflector (DBR) prompts the emitted light to radiate upward. The light extracted from the transparent sapphire propagates toward the Al film. When airflow is applied, the PDMS membrane together with the Al film is deformed and modulates the amount of reflected light reaching the PD. The light is absorbed by the InGaN layer in the PD, and the converted photocurrent signals can be used to indicate the airflow change.

**Fig. 2:**
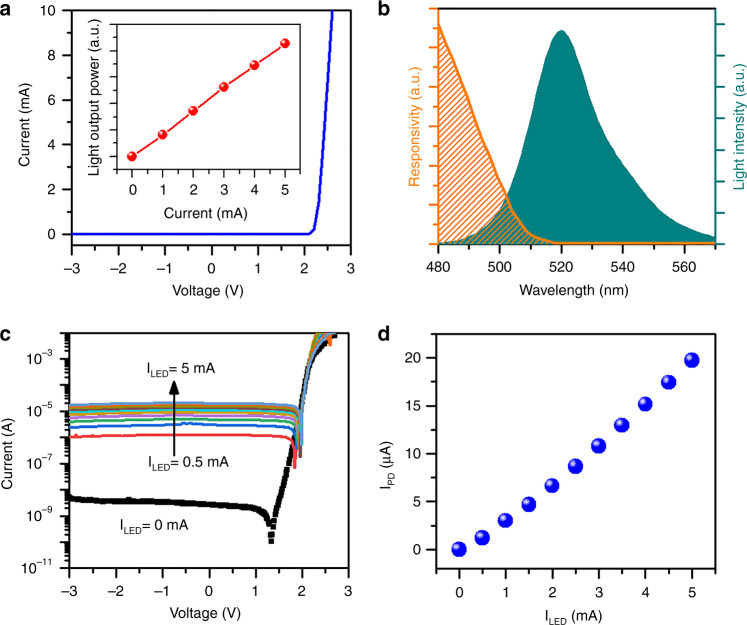
Optical and electrical properties of the GaN chip. **a** *I*–*V* characteristics of the LED. Inset showing a graph of the light output power as a function of the current. **b** EL spectra of the biased LED and spectral responsivity of the unbiased PD. **c** *I*–*V* curves of the PD measured under different LED currents. **d** Plot of the PD photocurrent as a function of the LED current

Formed on a single chip containing an InGaN/GaN multiquantum well (MQW), the LED and PD are the core elements responsible for light emission and detection, respectively, and their optical and electrical properties are characterized. Fig. 2a shows the current–voltage (*I*–*V*) curve of the LED measured using a source measurement unit (Keithley 2450), which provides a current resolution of 50 pA. The forward-biased voltage is 2.44 V at 5 mA, and the resistance calculated from the inverse slope of the *I*–*V* curve is 33.2 Ω. The inset of Fig. 2a reveals that the light output power increases with the bias current. With an identical InGaN-based MQW diode structure for photodetection in the PD, the response of PD to the LED emission is then verified. From the electroluminescence (EL) spectra of the LED plotted in Fig. 2b, the peak wavelength and spectral width are 521.5 and 26.8 nm at an LED current of 5 mA, respectively. It can be seen from the same figure that the responsivity curve of PD decreases as the wavelength increases and overlaps with the emission spectrum of approximately 30 nm. The observation of the partial spectral overlap is attributed to the absorption edge shift related to the quantum-confined stark effect and the band tail effect arising from the indium fluctuation of the MQWs^37^. Measured under no-airflow conditions, the *I*–*V* curves of the PD under different LED currents are plotted in Fig. 2c. In the absence of LED illumination, the photocurrents measured at reverse bias voltages are at a low level, on the order of 10^−9^ A. When an injection current of 5 mA is applied to the LED, the PD photocurrent rises significantly, by more than 5 orders of magnitude to 10^−5^ A. As observed from the current-current plot in Fig. 2d, a linear proportional relationship suggests that the photocurrent can highly respond to the intensity change of the LED emission.

**Fig. 3:**
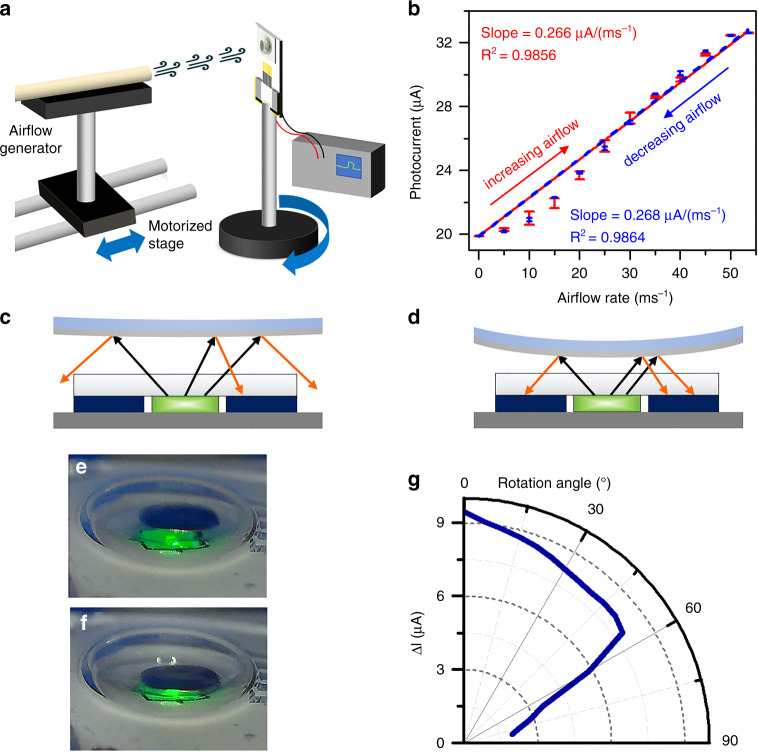
Response of the optical sensor to airflow. **a** Schematic diagram showing the setup for measuring the sensor response to airflow. **b** Plot of the photocurrent as a function of the airflow rate. Schematic illustrations of the distribution of reflected light under **c** low and **d** high airflows. Optical images of the sensor were taken under **e** low and **f** high airflows. **g** Polar plot showing the photocurrent response of the sensor at varying rotation angles

The photocurrent responses of the device at different airflow rates are acquired, and the measurement setup is illustrated in Fig. 3a. The inlet of the air pipe is connected to an airflow generator, and its outlet is mounted on a linear motorized stage. By controlling the position of the pipe outlet, the airflow experienced by the sensor can be adjusted. The airflow rate readings are calibrated using a commercial airflow meter (Taicang Huayu, WS200B). During the measurement, the LED is biased at a constant current of 5 mA, and the PD remains zero biased. Fig. 3b plots the measured photocurrents at different airflows of up to 53.5 ms^−1^. By performing linear regression on the data, we determine that the slopes of the fitted lines for increasing and decreasing airflow are 0.2664 and 0.2686 μA/(ms^−1^), respectively, and both R-squared values are larger than 0.98. Under low airflow, the PDMS membrane deforms less and is highly separated from the chip, as illustrated in Fig. 3c. With the divergence characteristics of the LED emission, the large spacing reduces the portion of reflected light reaching the PD. When increasing the airflow rate, the PDMS membrane together with the Al film is pushed toward the chip, resulting in more light being received by the PD, as depicted in Fig. 3d. Under the maximum detectable airflow of 53.5 ms^−1^, the PDMS membrane together with the Al film is very close to the chip surface. The photocurrent remains unchanged when further increasing the airflow beyond 53.5 ms^−1^. Furthermore, it can be seen from the captured image shown in Fig. 3e, f that the PDMS membrane deforms more at higher airflow. It is worth noting that there exists a stable background photocurrent of approximately 20 μA that does not respond to the applied airflow. This background signal originates from the light guided inside the chip rather than the externally reflected light. With the refractive index contrast of the sapphire/air interface at the chip surface, the emitted light with an angle of incidence larger than the critical angle of approximately 34.4° undergoes total internal reflection and is partially coupled into the PD through the transparent sapphire.

To investigate the effects of different airflow directions on the photocurrent response, the sensor is mounted on a motorized rotation stage. The airflow rate is fixed at 38.7 ms^−1^, and the photocurrents are collected from 0° to 80° with a step size of 10°. From the results plotted in Fig. 3g, it is observed that the photocurrent decreases gradually from 9.44 μA to 7.88 μA when the sensor is rotated from 0° to 80°. When the angle is further increased to 80°, the photocurrent change drops rapidly to 1.95 μA, suggesting that the sensor is less sensitive with increasing rotation angle.

**Fig. 4:**
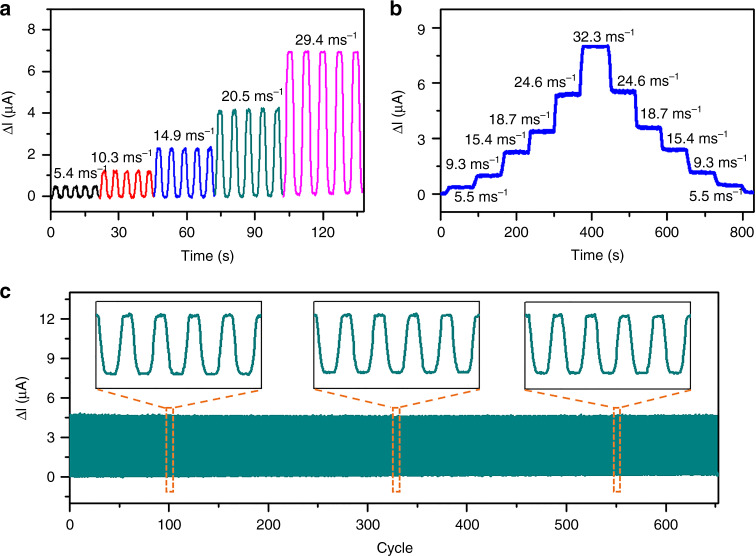
Dynamics responses of the sensor. Measured photocurrent response of the sensor measured under **a** continuous and **b** instantaneous airflow changes. **c** Reliability measurement of the sensor at 650 cycles at a frequency of 0.2 Hz

The dynamic responses of the sensor are studied by acquiring the photocurrent variation with time under different airflow conditions. Figure 4a shows the photocurrent response when the airflow rate is repeated in the range of 5.4–29.4 ms^−1^, indicating that the sensor exhibits a high degree of repeatability. The sensor response is then investigated by continuously increasing and decreasing the airflow between 5.5 and 32.3 ms^−1^, and the stepwise profiles of the photocurrent are observed in Fig. 4b. In addition, the reproducibility and stability of the sensor are evaluated by cyclic tests at a frequency of 0.2 Hz. As shown in Fig. 4c, the output photocurrent signals are highly stable over 650 cycles, and the photocurrent waveforms in different periods are consistent.

**Fig. 5:**
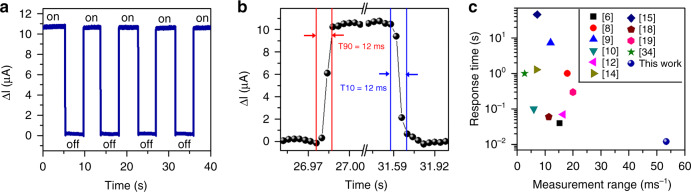
Response time of the sensor. **a** Temporal responses of the sensor. **b** Enlarged view of the temporal response showing the response time and recovery time. **c** Comparison of the response time and the measurement range with those of other reported airflow sensors

To analyze the temporal response of the sensor, the PD is connected to a current amplifier (SR570, Stanford Research Systems) and an oscilloscope (MDO32, Tektronix), and the LED current remains fixed at 5 mA. Figure 5a displays the transient response in the photocurrent when the airflow rate of 43.5 ms^−1^ is turned on and off. From the enlarged plot shown in Fig. 5b, the response time T90 and recovery time T10, defined as the time taken to reach 90 and 10% of the steady levels, are determined to be 12 ms. Highly similar transient responses are also observed at other airflow rates (see Supplementary information).

Figure 5c summarizes the response time and measurement range of the reported airflow sensors. Compared with the sensors with response times below 1 s, the sensor in this work provides a wider measurement range up to 53.5 ms^−1^. It is worth noting that the measured response time (12 ms) is more than three times shorter than the shortest value (40 ms) previously reported in the literature^6^. This remarkable performance can be attributed to the fast photoelectric conversion in the InGaN/GaN MQW diode structure, and the transient response to the electrical pulse signal is as low as 1.2 μs (see Supplementary Information), which is 2–3 orders of magnitude smaller than that of the previously reported sensor (at submillisecond scales)^11,13^.

**Fig. 6:**
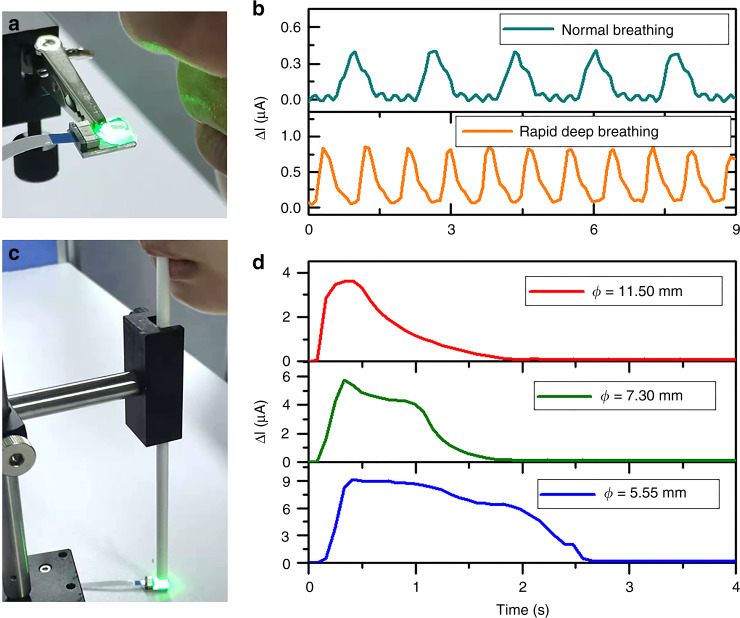
Measurement of human exhaled airflow. Optical images show a person exhaling through the **a** nose and **c** mouth with the aid of a tube to the sensor. The photocurrent response of **b** nose breathing and **d** mouth blowing to the sensor through tubes with varying diameters (ø) of 11.50, 7.30, and 5.55 mm

The proposed airflow sensor provides the attractive features of compact size, fast response, and wide detectable range and has a high potential for in situ measurement applications. For instance, airflow exhaled from the nose and mouth is considered a useful parameter for assessing human health^38,39^. To demonstrate its applicability in this area, the sensor is placed close to a person’s nose, with a separation of ~5 cm, as shown in Fig. 6a. From the plot in Fig. 6b, the periodic change in photocurrent related to the respiratory rate is observed, and the signal varying in the range of 0.4 μA implies that the airflow generated by nasal exhalation corresponds to a low airflow level. Under fast and deep breathing, a dense periodic photocurrent signal with a change of up to 8 μA can be obtained. In addition to normal breathing patterns, determining the peak flow rate of a person’s exhalation indicates how air is being expelled from the lungs, thereby judging the condition of asthma^40,41^. Figure 6c shows the airflow measurement of a person exhaling air through a tube. The photocurrents from the sensor incorporating tubes with ø = 11.50, 7.30, and 5.55 mm are plotted in Fig. 6d. Obviously, the photocurrent rises rapidly to the maximum value and decreases over time, representing a single continuous exhalation. It is found that a smaller ø results in a broader decay curve. The tube with the smallest ø of 5.55 mm can concentrate the airflow to the sensor, leading to the maximum photocurrent change of ~9 μA.

## Conclusion

In summary, we present the development of an optical airflow sensor based on the integration of a GaN chip with a PDMS membrane in a controllable and scalable manner. Flexible PDMS highly responsive to airflow can modulate the light emitted from the chip without the aid of external optics, and the detected photocurrent signals can reflect changes in airflow. The proposed airflow sensor exhibits outstanding performance in terms of response time and detectable range, making it suitable for in situ measurement in a wide range of practical applications.

## Materials and methods

### Fabrication of GaN chip

Six-micrometer-thick epitaxial structures consisting of 9 pairs of InGaN(2.5 nm)/GaN(12.5 nm) MQW are grown by metal–organic chemical vapor deposition on a 4-in. c-plane sapphire substrate. A square mesa of 1000 × 1000 μm^2^ and a hexagonal mesa with a side length of 155 μm are defined as a photodetector (PD) and light-emitting diode (LED), respectively, by photolithography. The unmasked GaN areas are etched to expose n-GaN using an inductively coupled plasma (ICP). To establish electrical isolation between the on-chip devices, a 10-μm-wide GaN region between the LED and PD is entirely removed by photolithography and ICP etching, followed by the deposition of a SiO_2_ passivation layer. The DBR acting as a bottom mirror is deposited by an optical thin-film coater. The p-pad and n-pad are deposited by electron-beam evaporation. After lapping and polishing of the sapphire face of the wafer, the chip with a size of 1 × 1 × 0.2 mm^3^ is diced by laser micromachining and then bonded on an aluminum-based PCB package. A detailed description of the process flow is provided in the Supplementary Information.

### Preparation of the PDMS membrane

The process begins with the use of a micropipette to dispense drops of 40 μL of deionized water into a 1-in. plastic petri dish. Because of the inherent hydrophobicity of the dish, the water droplets form a discrete hemispherical shape. The PDMS gel (Dow Sylgard 184) is prepared by mixing elastomer base and curing agent at a ratio of 10:1, followed by a degassing process in a vacuum chamber. The 1.35-g PDMS gel is poured over the droplets and allowed to rest for 10 min. As the PDMS gel with lower density is immiscible with water, the coated PDMS self-flattens over time and forms a thin membrane geometry above the water drops. The sample is then loaded in an oven at 80 °C for 2 hours for curing. The diameter and height of the PDMS circular cavity structure are 4.02 and 0.39 mm, respectively, and the membrane thickness is 0.01 mm. An Al reflective film with a diameter of 2 mm and a thickness of 0.01 mm is then formed by shaping the aluminum foil using a hole puncher.

## Supplementary information

Supplementary information - Marked Up

Supplementary information

## Data Availability

The data supporting plots within this paper and other findings of this study are available from the corresponding author upon request.
