# Supplementary material for: Chip-scale optical airflow sensor
Source: Microsyst Nanoeng. 2022 Jan 4;8:4. doi: 10.1038/s41378-021-00335-1 (PMC8724291; doi:10.1038/s41378-021-00335-1)
Supplement: Supplementary file 1 — Supplementary information - Marked Up [file 41378_2021_335_MOESM1_ESM.docx]

**Supplementary Information**

**Chip-Scale Optical Airflow Sensor**

**Yumeng Luo^1^, Xiaoshuai An^1^, Liang Chen^1^, and Kwai Hei Li^1,2,3,*^**

*^1^School of Microelectronics, Southern University of Science and Technology, Shenzhen 518055, China*

*^2^Engineering Research Center of Integrated Circuits for Next-Generation Communications, Ministry of Education, Southern University of Science and Technology, Shenzhen 518055, China*

*^3^Engineering Research Center of Three Dimensional Integration in Guangdong Province, Southern University of Science and Technology, Shenzhen 518055, China*

^*^Authors to whom correspondence should be addressed. Electronic mail: khli@sustech.edu.cn.

**S1 Fabrication process flow of GaN chip**


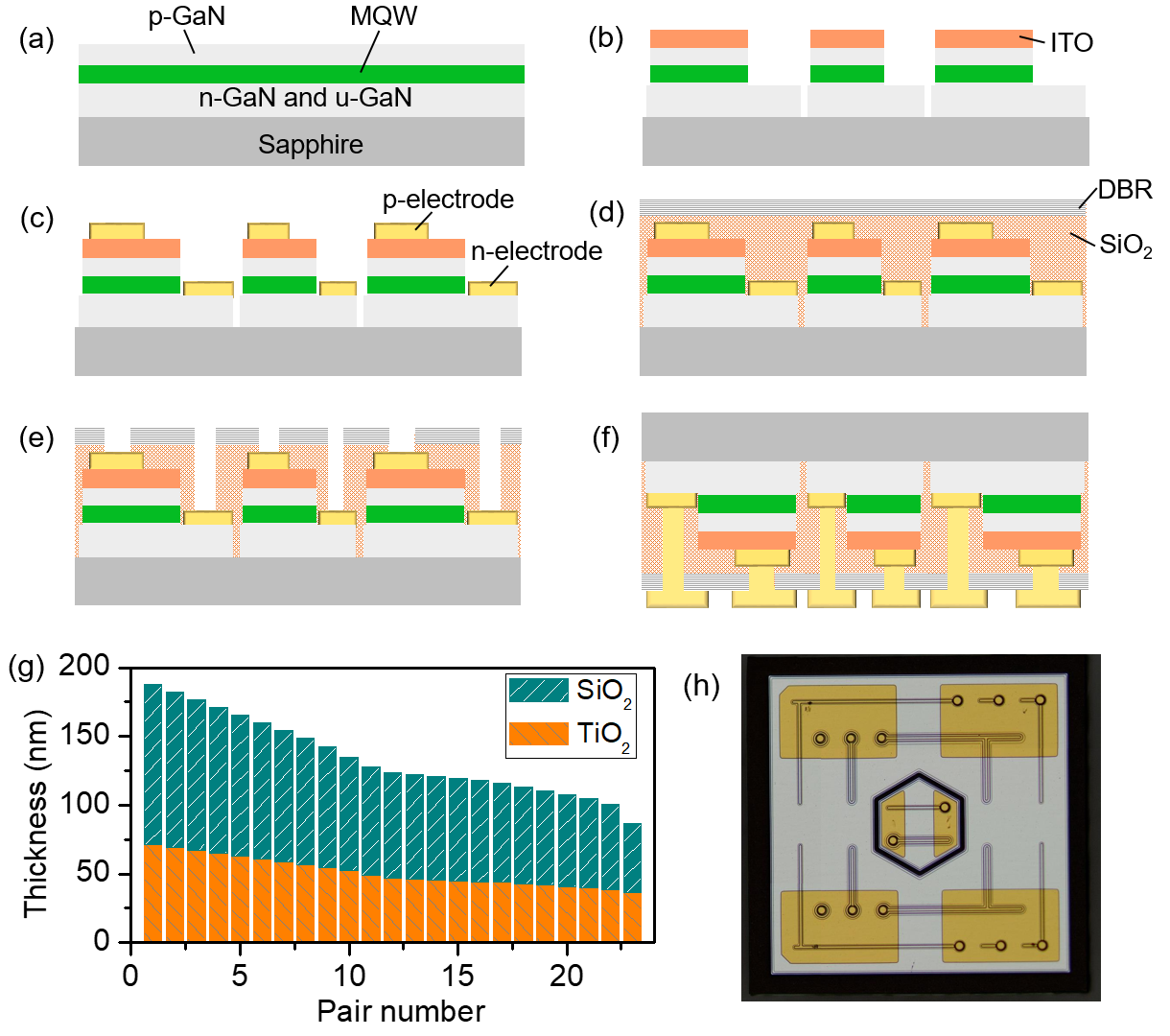


**Figure S1.** Schematic illustrations of the fabrication procedure of the GaN chip. (a) The starting GaN/sapphire wafer. (b) Definition of LED and PD mesa by photolithography and ICP etching, followed by deposition of ITO. (c) Electrical isolation of LED and PD by ICP etching, followed by deposition of electrodes by e-beam evaporation. (d) Deposition of SiO_2_ passivation layer and DBR. (e) Exposure of p-electrodes and n-electrodes by photolithography and ICP-etching. (f) Deposition of metal bond pads. (g) Plot of thickness distribution of 23 pairs of the SiO_2_/TiO_2_ DBR. (h) Microphotograph of the GaN chip.

A 6-μm-thick epitaxial structure composed of an unintentionally doped GaN (u-GaN), Si-doped n-GaN, InGaN/GaN multi-quantum well, and Mg-doped p-GaN is grown on a 4-inch sapphire substrate by metal-organic chemical vapor deposition, as shown in Figure S1(a). The microfabrication process begins with the mesa formation of the light-emitting diode (LED) and photodetector (PD) by photolithography and inductively coupled plasma (ICP) etching. A 0.12-μm-thick indium-tin-oxide (ITO) acting as a current spreading layer is coated on the p-GaN surface, as illustrated in Figure S1(b). To establish electrical isolation between the on-chip devices, a 10-μm-wide GaN interval between LED and PD is entirely removed by photolithography and ICP etching. The electrodes patterns are then photolithographically defined, followed by e-beam evaporation of a 1.65-μm-thick (Cr/Al/Ti/Pt/Au) as p-electrodes and n-electrodes on the exposed ITO and n-GaN, respectively, as shown in Figure S1(c). By means of plasma-enhanced chemical vapor deposition and optical thin film coater, a 0.36-μm-thick SiO_2_ passivation layer and a 3.16-μm-thick distributed Bragg reflector (DBR) containing 23 pairs of SiO_2_/TiO_2_ are respectively deposited on the sample, as described in Figure S1(d). After exposing the p-electrode and n-electrodes by photolithography and ICP etching, a 1.65-μm-thick Cr/Al/Ti/Pt/Au as p-pads and n-pads are e-beam deposited, as illustrated in Figures S1(e,f). The sapphire substrate is lapped and polished to a thickness of 200 μm. During the deposition process of DBR, the thickness of each SiO_2_/TiO_2_ pair is adjusted based on the reflectance feedback signals, resulting in an inhomogeneous thickness distribution of the SiO_2_/TiO_2_ pair. The thickness of each SiO_2_/TiO_2_ pair varies from 187.9 nm to 86.7 nm, as shown in Fig. 3(a). The processed wafer is diced into chips by laser micro-machining. A top-view microphotograph of the fabricated chip is shown in Fig. 1(g).

**S2 Transient response of GaN chip to electrical pulse signal**


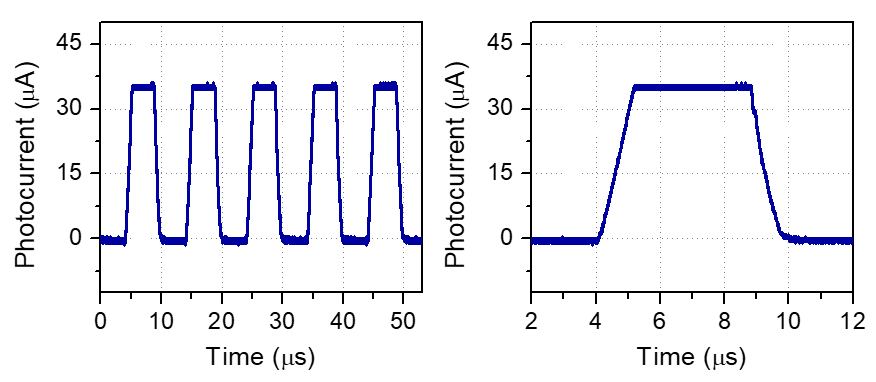


**Figure S2**. (a) Transient response of the GaN chip to electrical pulse signal. (b) Enlarged plot showing the rise time and fall time.

To investigate the transient response of the GaN chip, square-wave electrical pulses with a frequency of 10 kHz is applied to the LED and the photocurrent response of PD is measured using a current amplifier (Stanford Research Systems SR570) and an oscilloscope (Tektronix MDO32). Figure S2(b) plots the enlarged view of Figure S2(a), showing that both rise time and fall time are around 1.2 μs.

**S3 Transient response of the sensor under different airflow rates**


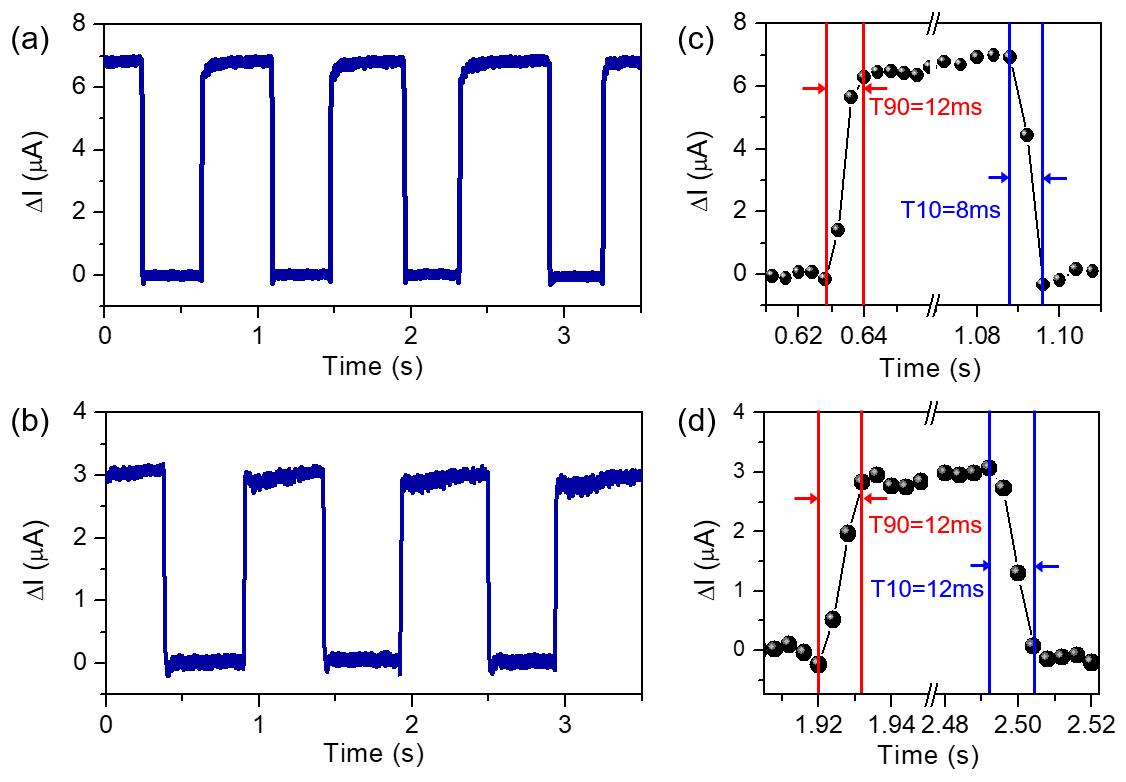


**Figure S3**. Temporal responses of the sensor under instantaneous airflow rates of (a) 28.5 ms^-1^ and (b) 17.1 ms^-1^. (c)-(d) Corresponding enlarged views of (a)-(b).

Figure S3(a) and (b) show the transient responses of the sensor when the instantaneous airflow rates of 28.5 ms^-1^ and 17.1 ms^-1^ are applied. From the enlarged plots in Figure S3(c) and (d), the response times are highly similar, which are around 12 mA.

**S4** **Comparison with previously reported airflow sensors**

| Materials | Sensor type | Meas.^a^ range (ms^-1^) | Linearity | Res.^b^ time (s) | Ref. ^c^ |
| --- | --- | --- | --- | --- | --- |
| Silicon nanowire | Piezoresistance | Up to 15.3 | Linear | <0.04 | [6] |
| MEMS cantilevers and anodic alumina membranes | Piezoresistance | 2-20 | Linear | >1 | [8] |
| Polyvinylidene difluoride fiber | Piezoelectric | 2-14 | Nonlinear | 7.392 | [9] |
| Platinum films on polyimide | Thermoresistance | 0-6 | Nonlinear | 0.1 | [10] |
| micro-wire-bonded hair-like  hot-wire | Thermoresistance | 1-17.5 | Nonlinear | 0.07 | [12] |
| Carbon nanotubes on silk fabric | Electrical resistance | 0.05-7 | Nonlinear | 1.3 | [14] |
| Graphene Oxide Film | Electrical resistance | 0.026-7.2 | Nonlinear | 46 | [15] |
| Polyethylene terephthalate film with silver nanoparticles | Magnetoelectric | 0.6-12 | Nonlinear | 0.06 | [18] |
| Triboelectrification-induced electroluminescence material and perovskite | Mechanoluminescence | 1-21 | Linear | <0.3 | [19] |
| GaN/Si | Optical | 0-2.779 | N/A | 1 | [34] |
| GaN/Sapphire and PDMS | Optical | 0-53.5 | Linear | 0.012 | This work |

^a^ Meas.: Measurement; ^b^ Res.: Response; ^c^ Ref.: corresponds to references listed in the manuscript.
